# Rice Biofortification With Zinc and Selenium: A Transcriptomic Approach to Understand Mineral Accumulation in Flag Leaves

**DOI:** 10.3389/fgene.2020.00543

**Published:** 2020-07-07

**Authors:** Faustino Adriano Roda, Isabel Marques, Paula Batista-Santos, Maria Glória Esquível, Alexis Ndayiragije, Fernando Cebola Lidon, B. P. Mallikarjuna Swamy, José Cochicho Ramalho, Ana I. Ribeiro-Barros

**Affiliations:** ^1^Ministério de Agricultura e Segurança Alimentar, Instituto de Investigação Agrária de Moçambique, Centro Zonal Noroeste, Lichinga, Mozambique; ^2^Universidade Eduardo Mondlane-Centro de Biotechnologia, Maputo, Mozambique; ^3^PlantStress&Biodiversity Lab, Forest Research Center (IM, JCR, AIRB) and Linking, Landscape, Environment, Agriculture and Food (PBS, MGE), Instituto Superior de Agronomia, Universidade de Lisboa, Lisbon, Portugal; ^4^International Rice Research Institute, Maputo, Mozambique; ^5^International Rice Research Institute, Laguna, Philippines; ^6^Unidade de Geobiociências, Geoengenharias e Geotecnologias, Faculdade de Ciências e Tecnologia, Universidade NOVA de Lisboa, Caparica, Portugal

**Keywords:** biofortification, flag leaves, rice, RNASeq, selenium, transcriptomics, zinc

## Abstract

Human malnutrition due to micronutrient deficiencies, particularly with regards to Zinc (Zn) and Selenium (Se), affects millions of people around the world, and the enrichment of staple foods through biofortification has been successfully used to fight hidden hunger. Rice (*Oryza sativa* L.) is one of the staple foods most consumed in countries with high levels of malnutrition. However, it is poor in micronutrients, which are often removed during grain processing. In this study, we have analyzed the transcriptome of rice flag leaves biofortified with Zn (900 g ha^–1^), Se (500 g ha^–1^), and Zn-Se. Flag leaves play an important role in plant photosynthesis and provide sources of metal remobilization for developing grains. A total of 3170 differentially expressed genes (DEGs) were identified. The expression patterns and gene ontology of DEGs varied among the three sets of biofortified plants and were limited to specific metabolic pathways related to micronutrient mobilization and to the specific functions of Zn (i.e., its enzymatic co-factor/coenzyme function in the biosynthesis of nitrogenous compounds, carboxylic acids, organic acids, and amino acids) and Se (vitamin biosynthesis and ion homeostasis). The success of this approach should be followed in future studies to understand how landraces and other cultivars respond to biofortification.

## Introduction

The 2030 Agenda of the United Nations brings forward 17 Sustainable Developing Goals among which agriculture lies at the core. However, according to the last report of the Food and Agriculture Organization (FAO et al., 2019), more than 820 million people in the world face hunger and undernourishment and, thus, poor health, particularly in Africa where greater efforts should be made to achieve the Zero Hunger by 2030. Undernutrition and micronutrient deficiencies account for three million deaths each year being more widespread problems than energy consumption (Prentice et al., 2008).

Zinc (Zn) and selenium (Se) are essential mineral nutrients (Fairweather-Tait et al., 2011). However, the intake of these minerals is deficient for ca. 30% (Zn) and 15% (Se) of the world population (White and Broadley, 2011; Moreno et al., 2013). Zn deficiency affects growth and the immune system, being especially severe for young children and pregnant women (White and Broadley, 2011). Se deficiency also weakens the immune system and has been associated with cardiovascular diseases, cognitive decline, cancer, and AIDS (Rayman, 2002; Jablonska and Vinceti, 2015). Thus, providing adequate contents of these minerals in food has become an important objective to fight “hiden hunger” in vulnerable populations (Hirschi, 2009). In this context, a supplementary and diversified diet is the most straight forward strategy to mitigate Zn and Se deficiencies. However, such strategies have been inefficient and uneffective to be implemented in developing countries were livelihoods are strongly dependent on small-scale agriculture (Hartikainen, 2005; Forsman et al., 2014). In order to overcome this constraint, biofortification offers a fast, reliable, and sustainable solution solution which has been successfully achieved in several crops, such as rice, wheat, and beans (White and Broadley, 2009, 2011; Li et al., 2018; Ramalho et al., 2020).

Rice is one of the world’s most important staple crops (FAO, 2016), constituting one of the most important sources of energy and micronutrients for more than half of the world population (Lucca et al., 2006; Muthayya et al., 2014). Both whole and polished rice grains contain low concentrations of Zn (6–28 mg kg^–1^) and Se (0.3 mg kg^–1^) (Walter et al., 2008), and agronomic biofortification has been used to increase these two minerals in the grain (Phattarakul et al., 2012; Ram et al., 2016; Mangueze et al., 2018; Lidon et al., 2017, 2019; Ramalho et al., 2020). Rice is also a model cereal species, and its genome was the first (among crops) to be fully sequenced in 2005 (Sasaki, 2005) and fully unified in 2013 (Kawahara et al., 2013). This crop has been thoroughly used to investigate the molecular mechanisms underlying several biological processes related to plant development, metabolism, and senescence from seedlings to grains (Jackson, 2016; Qian et al., 2018; Wang et al., 2018). The role of flag leaves in photosynthesis and nutrient mobilization to the grains has been highlighted by several earlier reports (Zhou et al., 2007; Pang et al., 2009; Sperotto et al., 2009; Tari et al., 2009; Xu et al., 2011). Thus, the identification of the molecular mechanisms involved in mineral transport from flag leaves to grains is of utmost importance to understand the biochemical processes associated with the absorption, translocation, and fixation of minerals, such as Zn and Se.

High−throughput *Omics* technologies such as *genomics*, *transcriptomics*, *proteomics*, *metabolomics*, *lipidomics*, or *interactomics* are nowadays widely used to understand biological systems as a whole (Sauer et al., 2007). Such extensive and integrated approaches allowed great advances in plant research, namely the elucidation of biological processes, such as plant development, plant-environment interactions, genomics-assisted breeding, or the discovery of phytocompounds with application in agriculture, medicine, and in a wide range of industries (Kole et al., 2015; Sundell et al., 2015; Bode et al., 2016; Nützman et al., 2016). Transcriptome analysis of rice flag leaves confirmed their importance in grain filling, namely, in the biosynthesis and translocation of photoassimilates and minerals to the seeds (Narayanan et al., 2007; Sperotto et al., 2009).

In this study, we have analyzed the transcriptional changes in flag leaves associated with the agronomic biofortification of rice with Zn and/or Se using a next-generation RNAseq approach that uses a high yield cultivar that is able to accumulate high levels of Zn and Se when exposed to biofortification treatments through foliar spraying (Mangueze et al., 2018).

## Materials and Methods

### Plant Material and Biofortification Experiments

Experiments were performed in the experimental fields of the International Rice Research Institute (IRRI), located at the Umbeluzi—Instituto de Investigação Agrária de Moçambique (IIAM) in Boane, Mozambique (Lat 26° 3′3.75″S; Long 32°21′56.48″E; Alt 8.8 m), using one rice cultivar, Makassane, containg 9.8 mg Kg^–1^ Zn and 0 mg Kg^–1^ Se in whole grains, under control conditions (that is, without any biofortification treatment with Zn or Se) (Mangueze et al., 2018). For the trials, three blocks of 95 m^2^ (15 × 5) each containing three biological replicates were established. Biofortification experiments were performed through single and combined foliar spraying of Zn and Se, using a back sprayer, at the beginning of grain filling, corresponding to Z51 stage in the Zadoks scale, i.e., at an adequate stage to promote the translocation to the grain (Cakmak, 2008; Cakmak et al., 2010). The following doses were applied: (i) 900 g ha^–1^ Zn (applied as zinc sulfate – ZnSO_4_ 7H_2_O), (ii) 500 g ha^–1^ Se (applied as sodium selenite – Na_2_O_3_Se), and (iii) 900 g ha^–1^ Zn together with- 500 g ha^–1^ Se. The Zn and Se doses as well as the use of Se-selenite (instead of Se-selenate) were based on previous reports in rice by Phattarakul et al. (2012), Lidon et al. (2019), and, especially, Mangueze et al. (2018), which used the same cultivars and cropped area. Each element was applied twice (using the same volume of the solution), with an interval of 7 days to reach the desired concentration. Control plants received only water.

The basal field fertilization was carried out with NPK (12:24:12) using 100 kg ha^–1^ 26 and 60 days after sowing. Foliar fertilization was made using 50 kg ha^–1^ of NPK (12:24:12) plus 50 kg ha^–1^ urea (46%) with a total of 200 kg ha^–1^ for the two applications.

For RNAseq analysis, flag leaves from three different plants per treatment were harvested 15 days after spraying, stored immediately in RNA Latter (Thermo Fisher Scientific), and frozen once in the lab.

### RNA Whole Transcriptome Deep Sequencing

Total RNA was extracted from 100 mg frozen material of each biological replicate per treatment using the inuPREP extraction Kit (Analytik Jena AG) following the manufacturer’s instructions. RNA integrity and purity were first evaluated by visual inspection of RNA bands through electrophoresis in a 1.5% agarose–TBE gel containing GelRed Nucleic Acid Gel Stain (Biotium) and then by an Agilent 2100 Bioanalyzer (Agilent). All samples had an RNA integrity number (RIN) higher than 8.9. Library preparation was performed with the TruSeq RNA Sample Prep Kit v2 (Illumina) and RNA-Seq analyzes by Illumina NovaSeq 6000 of 2 × 100 bp pair-end reads (30 million reads per sample) at Macrogen (South Korea).

### Alignment and Analysis of Illumina Reads

Raw reads obtained by sequencing were quality-checked using FastQC version 0.11.5 (Andrews, 2010). To reduce biases, artifacts as low-quality reads, adaptor sequences, or contaminant DNA were removed using Trimmomatic version 0.32 (Bolger et al., 2014). HISAT2 version 2.0.5 was used for the mapping of high-quality filtered reads against the reference genome (Os-Nipponbare-Reference-IRGSP-1.0) downloaded from the Rice Genome Annotation Project Database^1^ (Kawahara et al., 2013). Known genes and transcripts were assembled with StringTie version 1.3.3b (Pertea et al., 2015, 2016) based on the reference genome model. After assembly, the abundance of gene transcripts was calculated for each sample and normalized as FPKM (Fragments per Kilobase of transcript per Million Mapped reads) using Cufflinks version 2.2.1 (Trapnell et al., 2010). The similarity between samples was obtained through Pearson’s coefficient of the Log_2_(FPKM+1) value with a range of −1 ≤ r ≤ 1 (the closer the value is to 1, the more similar the samples are) and graphically depicted using a correlation matrix.

### Differentially Expressed Gene Analysis

During data preprocessing, low quality transcripts were filtered. Afterward, log2 transformation of FPKM+1 and quantile normalization were performed. To identify the differentially expressed genes (DEGs) from the dataset, an FDR adjusted *P*-value of <0.05 was set and a fold change (FC) of ≥2 was assigned. For significant lists, a hierarchical clustering analysis was performed to group the similarity of transcripts and samples by expression level of normalized values. Standardized expression patterns were visualized as Z-scores in a heatmap generated by hierarchical clustering (function hclust in R). The significant DEGs found were mapped on the 12 chromosomes of rice using the chromosome map tool in Oryzabase database (Yamazaki et al., 2010), and a map was drawn based on output generated. Following Raza et al. (2019), all DEGs with a gene ontology (GO) function related to cation ion binding/transport, heme binding, Se, Zn ion binding/transport, metal ion binding, transport, and homeostasis were filtered and considered as putative candidate genes (CGs) associated with traits of interest in biofortification.

### Functional Annotation, Enrichment and Pathway Analysis

To assign functional categories to the DEGs, a gene-set enrichment analysis was performed using the DAVID 6.7 database for annotation, visualization, and integrated discovery, an online tool for the analysis of the relevant biological annotation of gene lists^2^. The significant DEGs were annotated for GO terms and categorized into biological process (BP), molecular function (MF), and cellular component (CC). For significant DEGs, a gene enrichment test was then performed using the DAVID default background, representing the corresponding genes with at least one annotation in the analyzing categories in the enrichment calculation. *P*-value for enrichment was calculated for each GO term represented and corrected via Bonferroni family-wise error rate (FWER) method. Only the GO terms exhibiting a corrected *P*-value of <0.05 were considered to be significantly enriched for a given set of genes. To investigate which DEGs were activated or suppressed in different class of pathways, gene expression information was mapped using the Kyoto Encyclopedia of Genes and Genomes, KEGG^3^. Pathway images were generated using the online tool KEGG Mapper-Colour Pathway^4^. Raw and processed RNA-sequencing data have been deposited in NCBI.

## Results

### Overall Transcriptome Profiling and Mapping Statistics

**FIGURE 1:**
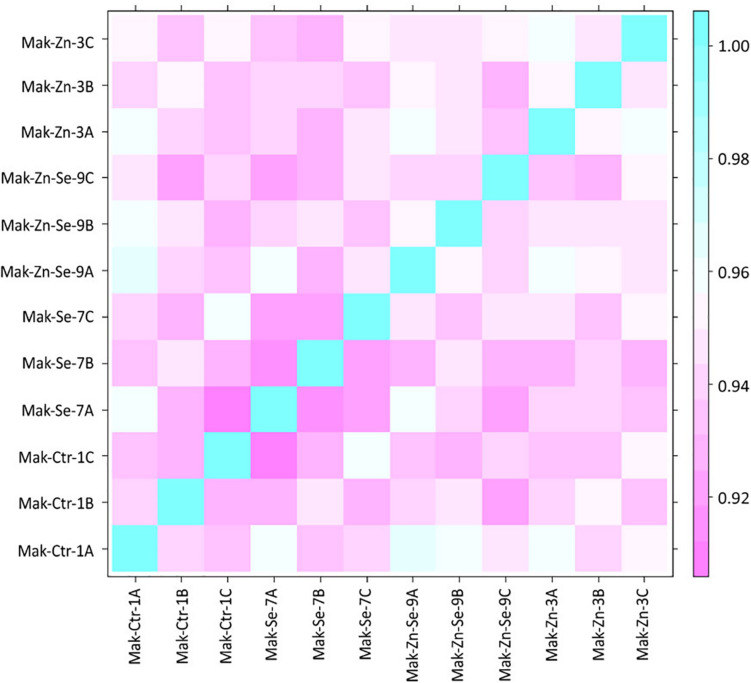
Correlation matrix for all samples obtained through Pearson’s coefficient of the Log2(FPKM+1) value. The closer the value is to 1, the more similar the samples are.

In total, the 24 RNA libraries generated an average of 38 million reads with a GC content of 53.28% (Table 1). An average of 1.64% of the reads were removed after being trimmed. The vast majority (98.36%) of the total number of reads was mapped to the reference rice genome demonstrating a high coverage over the transcriptome. From these, an additional average of 7.55% reads could not be mapped into the reference genome. Statistics of each sample are provided in detail in Supplementary Table S1. After trimming and cleaning, a total of 34 million reads were analyzed. A high similarity was found between samples through Pearson’s coefficient of the Log_2_(FPKM+1) value (Figure 1).

**TABLE 1:** Overview of RNA-Seq data of rice cultivar Makassane (Mak) in control conditions (Ctr) and under different treatments of Selenium (Se500: 500 g ha^–1^) and Zinc (Zn900: 900 g ha^–1^) or with these two elements together (Zn-Se).

Genotype	Setup	Total reads	GC (%)	Processed	Mapped	Unmapped
Mak	Ctr	36,821,836	54.03	36,205,938 (98.32%)	33,869,031 (93.55%)	2,336,906 (6.45%)
Mak	Se500	34,451,820	52.81	33,907,595 (98.42%)	30,784,505 (90.79%)	3,123,090 (9.21%)
Mak	Zn900	39,822,173	52.91	39,162,976 (98.36%)	36,396,575 (92.94%)	2,766,401 (7.06%)
Mak	Zn-Se	41,069,693	53.35	40,394,978 (98.42%)	37,382,433 (92.54%)	3,012,544 (7.46%)
**Average**		38,041,381	53.28	37,417,872 (98.36%)	34,508,136 (92.45%)	2,809,735 (7.55%)

*Numbers indicate the average of the three biological replicates concerning the total number of reads after trimming (total reads), GC content (GC%), number of cleaned reads after trimming (processed), number of reads mapped to the reference (mapped), and number of reads that failed to aligned (unmapped). Statistics of each sample are provided in detail in Supplementary Table S1.*

### Differentially Expressed Genes

**FIGURE 2:**
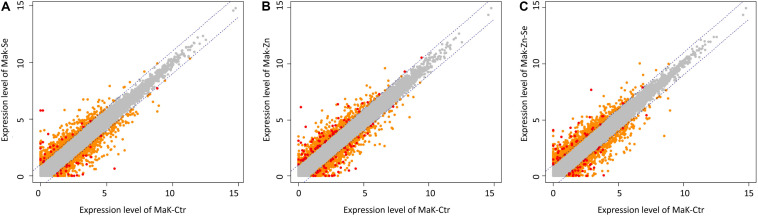
Scatter plots of expression levels between control and the average normalized value of the Zn-biofortified pool **(A)** between control and Se-biofortified pool **(B)** and between control and Zn-Se-biofortified pool **(C)**. Transcripts levels significantly above control levels are upregulated in response to biofortification while those below control levels are downregulated. Each plot contains all identified transcripts, including non-significant (gray dots) as well as specific DEGs that are considered significant under |FC| ≥ 2 (orange dots) and under |FC| ≥ 2 and *P* < 0.05 (red dots).

**FIGURE 3:**
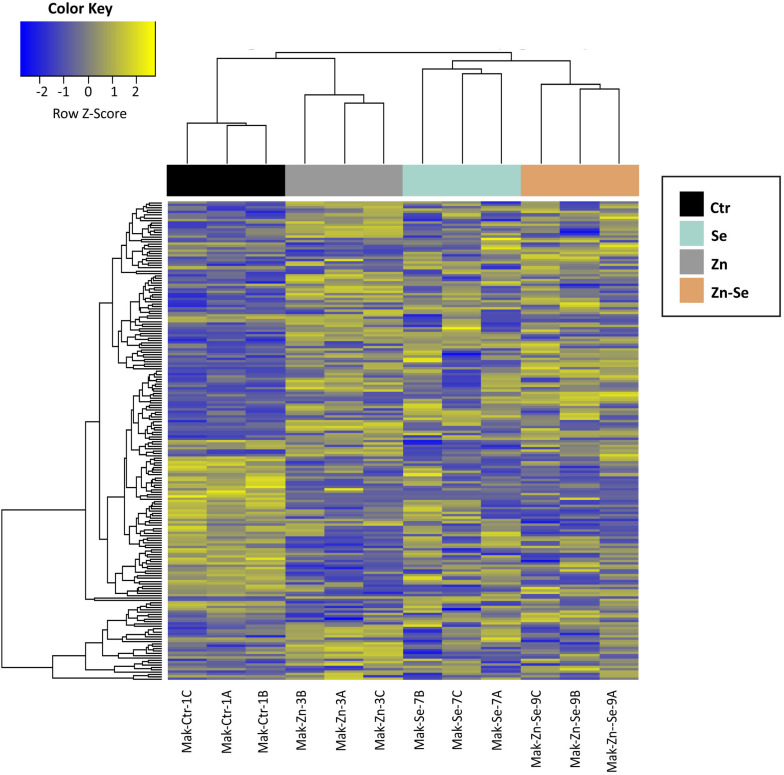
Heatmap of rice cultivar Mak differentially expressed genes after biofortification with single and combined Zn and Se and application. Expression values are depicted as Z-standardized scores for each DEG, where blue represents downregulated DEGs and yellow upregulated DEGs.

We identified 3170 genes that were differentially expressed between biofortified and control plants, of which only 224 were significantly different (FDR < 0.05) and had a FC ≥2 (Figure 2). Hierarchical clustering analysis of all DEGs showed no specific trends in expression convergence (Figure 3), suggesting that biofortification with Se, Zn, and Se-Zn led to different changes in DEGs.

**FIGURE 4:**
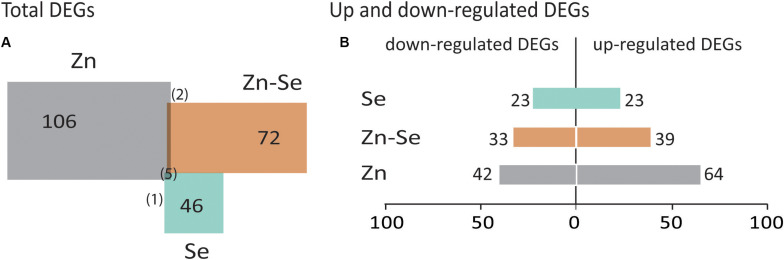
Total number of differentially expressed genes (DEGs). Common DEGs between comparisons are indicated between brackets **(A)**. Up- and downregulated DEGs between comparisons with | FC| ≥ 2 and *p* < 0.05 **(B)**. Comparisons indicate the number of DEGs found in biofortified flag-leaves with Se, Zn, or Zn-Se in comparison with control conditions. Colors of the biofortified pools are the same as in Figure 3.

Biofortification with Zn alone triggered a higher number of DEGs (106) than in combination with Se (72) and even less when only Se was used (46) (Figure 4A). DEGs were usually upregulated in the Zn-biofortified and the Zn-Se-biofortified pool (61 and 54%, respectively), while Se-biofortified flag-leaves expressed an equal number of up- and downregulated DEGs (23%) (Figure 4B and Supplementary Tables S2–S4).

The type of DEGs and the level of FC also varied between the three biofortified pools. For instance, distribution trends in terms of FC ranged from *ca.* −10 to 54 for DEGs in the Se-biofortified pool (Supplementary Table S2). Two genes were found to be highly enriched with FC 4–5 times higher than for the remaining genes: ataxin-2 C-terminal region family protein (Os03g0180300) and a CBL-interacting protein kinase 16 (Os09g0418000). Meanwhile, GFA2 (Os06g0116800) and a hypothetical protein (Os03g0180300) were the top downregulated genes. From those 46 genes, 7 were putative CGs associated with traits of interest in Se-biofortification: Biotin synthase (Os08g0540100), Similar to inducible alpha-dioxygenase (Os12g0448900), Queuine tRNA-ribosyltransferase (Os09g0469900), Thiazole biosynthetic enzyme 1^–1^ (Os07g0529600), Cytochrome P450 (Os02g0221900), Zinc finger (Os01g0667700), and Protein phosphatase 2C domain (Os05g0358500).

In comparison, FC of DEGs from flag leaves of Zn-biofortified plants varied between *ca*. −13 to 61 (Supplementary Table S3). Top downregulated included a Resistance protein candidate (Os05g0318700) and a Photosystem I protein-like (Os07g0148900) encoding genes, while a 2-oxoglutarate dehydrogenase E2 subunit gene (Os04g0394200) was found to be highly upregulated, with an FC six times higher than the remaining DEGs. From those 106 genes, eight were considered putative CGs associated with traits of interest in Zn-biofortification: NifU-like protein (Os01g0662600), WD-40 repeat containing protein (Os02g0791800), Laccase-6 (Os01g0850550), Ribose-phosphate pyrophosphokinase 3 (Os01g0723600), C-type lectin domain (Os01g0104000), Homodimeric diiron-carboxylate protein (Os04g0600300), Queuine tRNA-ribosyltransferase (Os09g0469900), and Cytochrome P450 (Os02g0173100).

In the set of plants biofortified with the two elements (Zn-Se), FC of DEGs varied between ca. -13 to 27 (Supplementary Table S4). A DRE binding protein 2 (Os01g0733801) was found to be the top downregulated gene, while a cystathionine gamma-synthase (CGS) (Os03g0376100) and an UDP-glucuronosyl/UDP-glucosyltransferase (UGT) family protein genes (Os01g0736100) were the top upregulated genes under Zn-Se biofortification. From those 72 genes, eight could be CGs associated with traits of interest in Zn-biofortification: Adaptin ear-binding coat-associated protein 2 (Os10g0476000), Endoribonuclease Dicer homolog 2a (Os03g0583900), Thioredoxin family Trp26 (Os01g0559000), Cytochrome family protein (Os11g0151400), Syntaxin 6 (Os08g0244100), Cytochrome P450 (Os02g0173100), Queuine tRNA-ribosyltransferase (Os09g0469900), and Secretory carrier membrane protein (Os04g0597000). Interestingly, one gene (Os03g0103300) from the Se-Zn biofortified pool was a quantitative trait loci (QLT) G-3-1 protein, targeted for low-temperature germinability (Supplementary Table S4).

Only six DEGs were shared between the three biofortified pools: Eukaryotic initiation factors 3 (Os04g0112300) and 4 (Os04g0112300), Cytochrome P450 (Os02g0173100 and Os02g0221900), UDP-glucuronosyl/UGT (Os01g0736100), and Queuine tRNA-ribosyltransferase (Os09g0469900). Additionally, an Ethylene response factor 2 gene (Os07g0617000) and two Hypothetical proteins (Os01g0358300 and Os07g0536966) were commonly upregulated in the Zn- and in the Se-biofortified pool (Supplementary Tables S2, S3). Genes encoding a Prolin-rich protein (Os04g0612500) and a Chitinase-like protein (Os09g0494200) (both downregulated) were found to be commonly expressed between the Zn- and the Zn-Se- biofortified pool (Figure 4B and Supplementary Tables S3, S4).

DEGs were unevenly distributed among the 12 rice chromosomes being predominant on chromosome 1 (with 32 DEGs), chromosome 3 (with 23 DEGs), and chromosome 4 (with 22 DEGs; Supplementary Figure S1). Few DEGs could be mapped on chromosomes 11 (3 DEGs, none from Se-biofortification) and 10 (5 DEGs) and on chromosome 12 (7 DEGs). DEGs from biofortification with Se, Zn, and Se-Zn were predominantly mapped on chromosome 1 (7 DEGs), chromosomes 1 and 4 (13 DEGs), and chromosome 1 (10 DEGs), respectively.

### Gene Ontology Annotation of DEGs

**FIGURE 5:**
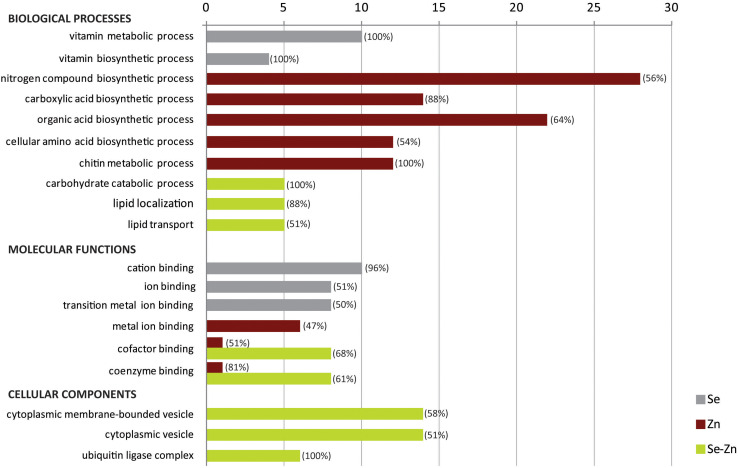
Gene ontology enrichment analysis of biological processes (BP), molecular function (MF), and cellular component (CC) for up- and downregulated genes between biofortified flag leaves with single and combined Zn and Se and application in comparison with control conditions. The percentage of upregulated DEGs found within each category is indicated between brackets.

Gene ontology categories from the list of significant DEGs were overall upregulated (although at different levels) except for “metal ion binding” in the Zn-biofortified pool (Figure 5). GO categories also showed opposite profiles in the three biofortified pools, which corroborates the differential gene regulations (Figure 5). BP such as “vitamin metabolic process” and “vitamin biosynthetic process” were enriched in the Se-biofortified pool while “nitrogen compound biosynthetic process,” “carboxylic acid biosynthetic process,” “organic acid biosynthetic process,” “cellular amino acid biosynthetic process,” and “chitin metabolic process” were the in the Zn-biofortified pool. In contrast, BP categories such as “carbohydrate catabolic process,” “lipid localization,” and “lipid transport” were enriched after Zn-Se biofortification. MF such as “cation binding,” “ion binding,” and “transition metal ion binding” were enriched after Se-biofortification. Categories as “metal ion binding,” “cofactor binding,” and “coenzyme binding” were enriched after Zn-biofortification, the two latter categories also enriched after biofortification with the two elements together (Figure 5). Cellular components (CC) such as “cytoplasmic membrane-bounded vesicle,” “cytoplasmic vesicle,” and “ubiquitin ligase complex” were only enriched after Zn-Se biofortification (Figure 5).

### Effect of Biofortification on Biological Pathways

**FIGURE 6:**
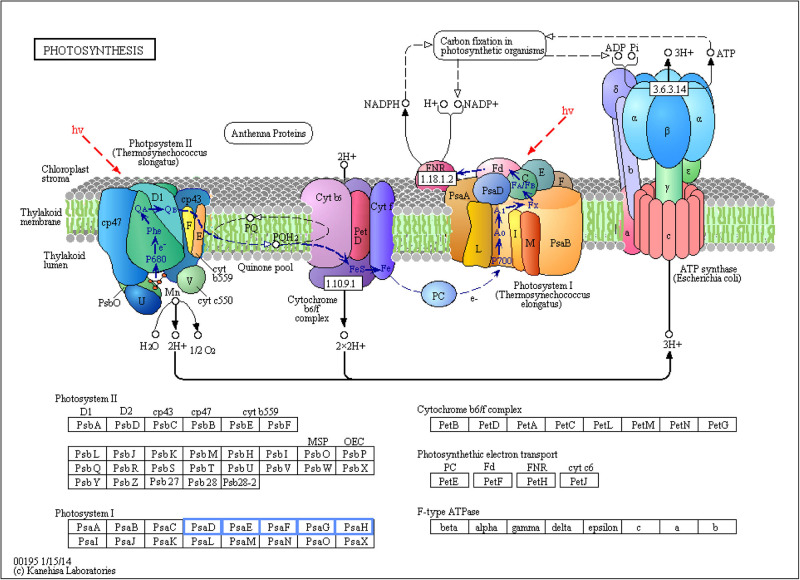
Significant differentially expressed genes (DEGs) involved in photosynthesis of Zn-biofortified rice cultivar Mak (*P* < 0.01). Genes significantly downregulated by Zn-biofortification are shown in blue boxes. White boxes indicate non-responsive genes.

**FIGURE 7:**
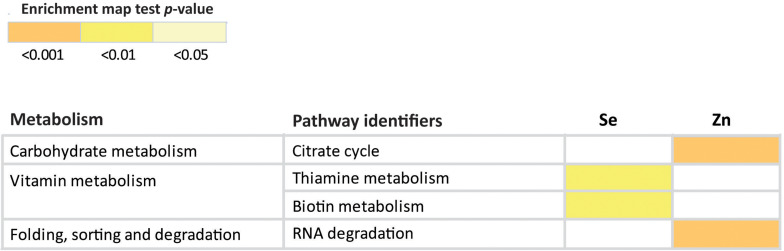
Heatmap showing significant KEGG pathways involving upregulated DEGs after Se and Zn biofortification. Colors indicate the level of significance.

Photosynthesis was the only biological pathway involving downregulation of gene expression and only in the biofortification with Zn. Five different genes, all involving Photosystem I (*PsaD*, *PsaE*, *PsaF*, *PsaG*, and *PsaH*), were significantly downregulated after Zn-biofortification (*P* < 0.01; Figure 6). By contrast, four different biological pathways involving upregulated DEGs were significantly enriched after biofortification: the citrate cycle (TCA cycle) from the carbohydrate metabolism and the RNA degradation pathway (respectively, *P* < 0.001 and *P* < 0.05), while the vitamin metabolic pathway involving the production of thiamine and biotin were enriched after Se-biofortification (*P* < 0.01 in both pathways; Figure 7).

**FIGURE 8:**
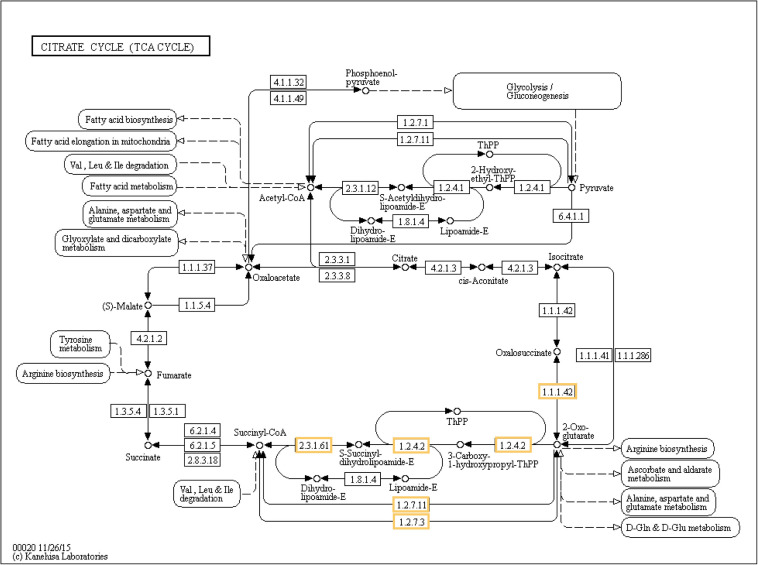
Significant differentially expressed genes (DEGs) involved in the TCA cycle of Zn-biofortified rice cultivar Mak (*P* < 0.001). Genes significantly upregulated by Zn biofortification are shown in yellow boxes. White boxes indicate non-responsive genes.

Six different upregulated genes were found to be enriched in the tricarboxylic acid (TCA) cycle: one isocitrate dehydrogenase (1.1.1.42), two 2-oxoglutarate dehydrogenase E1 component (1.2.4.2), 2-oxoglutarate dehydrogenase E1 component (2.3.1.61), and two 2-oxoglutarate/2-oxoacid ferredoxin oxidoreductase subunit alpha (1.2.7.11 and 1.2.7.3; Figure 8). Three genes associated with the pathway of RNA degradation were also found after biofortification with Zn: ATP-dependent RNA helicase DDX6/DHH1 (DDX6), enhancer of mRNA-decapping protein 3 (EDC3), and enhancer of mRNA-decapping protein 4 (EDC4) (Supplementary Figure S2). In comparison, the thiamine metabolism pathway was enriched after Se-biofortification involving the upregulation of nucleoside-triphosphatase (3.6.1.15), thiamine-monophosphate kinase (2.7.4.16), and adenylate kinase (2.7.4.3), while the biotin metabolism pathway was enriched after Se-biofortification directly through the upregulation of biotin synthase (2.8.1.6) (Supplementary Figure S3).

## Discussion

Rice is both a model plant species and one of the most important staple foods worldwide. It is, however, a poor source of micronutrients, such as Zn and Se, whose deficiency has several impacts on human health and child growth, particularly in developing countries. To compensate nutrient-poor staples, genetic and agronomic biofortification strategies have been widely used to fight hidden hunger (Kondwakwenda et al., 2018; Neeraja et al., 2018; Hefferon, 2019; Zhou et al., 2019). While genetic biofortification approaches are powerful tools for cereals (including rice) biofortification, the extended time frame and resources needed for conventional breeding as well as the economic, ethic, and legal issues associated with genetically modified crops are among the main limiting factors, and, in both cases, mineral availability is largely dependent on the soil properties (Cakmak, 2008). On the other hand, agronomic biofortification constitute a useful and reliable short-term strategy, complementary to conventional breeding (Cakmak, 2008; White and Broadley, 2009, 2011; Ramalho et al., 2020).

However, the transcriptional basis of the biofortification process at the leaf level is still poorly understood (Sperotto et al., 2013; Neeraja et al., 2018; Tang et al., 2018). In this context, we have analyzed the transcriptome of rice flag leaves to gain insight about their contribution to mineral mobilization to grains. For that we have used a rice cultivar (Makassane) with high-yield (6–7 tonnes per hectare), high grain quality, and resistance to the two major rice diseases (bacterial leaf blight and blast) that has been bred by the IRRI and adapted to the irrigated agro-ecological conditions of Mozambique, Southern Africa (Singh et al., 2013), that have a good perspective for future commercialization. Although a certain degree of Zn-Se antagonism has been referred in some rice cultivars, in this specific genotype co-application of Zn and Se did not interfere with Zn accumulation while also promoting Se accumulation in grains (Mangueze et al., 2018).

Transcriptome analysis of flag leaves from rice plants biofortified with Zinc (Zn; 900 g Zn ha^–1^), Selenium (Se; 500 g Se ha^–1^), and both minerals (Zn-Se; 900 g Zn ha^–1^ and 500 g Se ha^–1^) identified a total of 34 million reads, which is in line with the results obtained from other transcriptome studies on biofortified rice (Neeraja et al., 2018), wheat (Mishra et al., 2019), and maize (Yi et al., 2019). The number of DEGs was *ca.* 3000, which is also in agreement with other transcriptome projects in rice, where *ca*. 1000–3000 DEGs have been reported (e.g., Li et al., 2018; Cao et al., 2019; Chen et al., 2019; Sun et al., 2019). The response of the rice transcriptome is quite variable, depending on the organ, cultivar, and environmental conditions. For instance, in panicles of two Indian landraces (Chittimutyalu, CTT and Kala Jeera Joha, KJJ) and one improved variety (BPT 5204), with differential ability to uptake zinc, the total number of DEGs varied from *ca.* 1400 (BPT 5204) to *ca.* 2200 in CTT and KJJ, of which *ca.* 450–800 with significant FC (Neeraja et al., 2018). On the other hand, in roots of a Chinese rice cultivar (Nipponbare) subjected to Cadmium (Cd) stress ca. 1200 DEGs were identified, 226 with significant FC at low and 1162 at high Cd concentrations, 10 μM and 100 μM, respectively (He et al., 2015). In our study, the rice biofortification of cultivar Makassane triggered a relatively low number of DEGs (224) with significant FC. Such a low number might be anchored in the following arguments: (i) molecular changes associated with the biofortification processes are narrow and limited to specific pathways; and/or (ii) slight (statistically non-significant) changes in gene expression are enough to trigger the pathways involved in mineral absorption and translocation; and/or (iii) post-transcriptionally regulated events.

The number of significant DEGs was higher in flag leaves of Zn-biofortified plants (106) than those from Se- and Zn-Se-biofortified plants (72 and 46, respectively) (Figure 4). Differences in DEGs were extensive to FC (−10 to 54 for Se, −13 to 61 for Zn, and −13 to 27 for Zn-Se) as well as to the gene classes (Supplementary Tables S2–S4). These differences are in line with the reported variation of the rice transcriptome referred above (He et al., 2015; Neeraja et al., 2018) and might be also related to the fact the mineral concentrations used for spraying were higher for Zn than for Se, triggering greater changes related to more demanding changes during Zn accumulation.

Gene ontology analysis of DEGs showed also different profiles of the three sets of biofortified samples, corroborating the expression patterns of the individual set of DEGs (Figure 5). Thus, while, in the case of Se biofortification, vitamin biosynthesis and metabolism were among the main BP, in the case of Zn, the main BP included biosynthesis of nitrogen compounds, carboxylic acids, organic acids, amino acids, as well as chitin metabolism. Additionally, in the case of the combined Zn and Se application, the BP associated with carbohydrate metabolism and lipid dynamics were also enriched. Such distinct GO profiles are likely related to the specific roles of Se and Zn in plant functioning as well as with specificities of Zn and Se biofortification processes, even considering a certain antagonism extent between these two minerals as regards their accumulation potential (Boldrin et al., 2012; Mangueze et al., 2018). This hypothesis is supported by the classification based on the MF, i.e., ion homeostasis in Se-biofortified flag leaves, probably related to the role of this mineral in controlling oxidative stress, the coenzyme/cofactor function of Zn in Zn-biofortified flag leaves, and a rather diverse set of GOs in the double biofortified flag leaves (Sunde, 2018). Nevertheless, the three biofortification processes have in common alterations in cell metabolism inherent of the mineral mobilization and their specific cellular functions (Garg et al., 2018).

Among the top DEGs, two genes encoding an ataxin-2 and a CBL (Calcineurin B-like)-interacting protein kinase (CIPK) were the most abundant in Se-biofortified flag leaves. Ataxin-2 is a CID (CTC-interacting domains) protein highly conserved among eukaryotes (Jiménez-López and Guzmán, 2014). Although the functional characterization of ataxins in plants has not yet been fully addressed, key and evolutionary conserved roles of this group of proteins have been proposed, namely, in post-transcriptional regulatory assembly in many biological processes, including, growth, development, and environmental responses (Jiménez-López et al., 2015; Ostrowski et al., 2017). Thus, it is possible that Se-biofortification in rice is highly regulated post-transcriptionally through Ataxin-2, explaining the low number of DEGs identified in this pool of samples. On the other hand, the activation of the plant-specific CBL-CIPK complex is likely related to the induction of a plant stress signaling cascade (Ligaba-Osena et al., 2017; Zhang et al., 2018; Aslam et al., 2019; Liu et al., 2019) necessary to mobilize Se. In fact, although its role in plants remains controversial, Se is known to act in mechanisms of plant protection against a variety of abiotic stresses, such as cold, drought, desiccation, and metal stress (Gupta and Gupta, 2017). Furthermore, several studies have also found that CBL-CIPK pathways work as regulators in nutrient transport systems, namely, Na^+^ (Shi et al., 2002), K^+^ (Xu et al., 2006), Mg^2+^ (Tang et al., 2015), and NO_3_^–^ (Ho et al., 2009).

With Se biofortification, the induction of vitamin metabolic pathways related to the production of the thiamine and biotin were enriched (Figure 7), the first involving the transcriptional activation of genes encoding a nucleoside-triphosphatase, a thiamine-monophosphate kinase, and an adenylate kinase and the second the upregulation of a biotin synthase (Supplementary Figure S3). Consequently, biofortification with Se might reinforce thiamine presence in rice, which is usually very poor, ranging from 0.053 to 3 mg per 100 g of grain. These values become even lower with the elimination of the aleurone layer (where thiamine is predominantly stored) in polished rice as well in cooked rice (Minhas et al., 2018).

With regards to the flag leaves from Zn-biofortified plants, the most abundant transcript corresponded to a gene enconding a 2-oxoglutarate (2-OG) dehydrogenase (2-ODD), the largest family of non-heme oxidizing enzymes (Kawai et al., 2014), with a key function in the TCA cycle that uses 2-OG as an obligatory substrate (Scheible et al., 2000). This will link the TCA cycle (and thus ATP production) to amino acid, glucosinolate, flavonoid, alkaloid, and gibberellin biosynthesis (Araújo et al., 2014). Taking into account the wide Zn roles in plants, which include the composition of proteins and other macromolecules, its action as a functional, structural, or regulatory cofactor of a large number of enzymes, and its role in gene expression control (Brown et al., 1993), the huge activation of 2-ODD might be related to the enhanced cell metabolic activity induced by Zn biofortification. Such large accumulation of 2-ODD transcripts is also in line with the GO analysis and its involvement in several biosynthetic pathways (Araújo et al., 2014; Farrow and Facchini, 2014; Wang et al., 2019).

In line with the GO analysis and with the proposed roles for Zn in plant cells, the analysis of the impact of Zn biofortification in biological pathways confirms the relation with the TCA cycle and carbohydrate metabolism but also with RNA degradation, highlighting its role in cell metabolism and control of gene expression (Brown et al., 1993). The genes encoding enzymes from the TCA cycle included an isocitrate dehydrogenase, three 2-oxoglutarate dehydrogenases, and two 2-oxoglutarate/2-oxoacid ferredoxin oxidoreductases involved in plant defense against stress (Araújo et al., 2014; Yu et al., 2018). The genes associated with RNA degradation included an ATP-dependent RNA helicase (DDX6), and two enhancers of mRNA-decapping proteins 3 and 4 (EDC3 and EDC4), factors regulating mRNA turnover in plants probably involved in developmental processes (Goeres et al., 2007) as well as in stress responses (Kawa and Testerink, 2017).

On the other hand, a Photosystem I (PSI) gene was among the top downregulated DEGs. A deeper analysis of biological pathways indicates that Zn biofortification resulted in a downregulation of several photosynthesis-related genes, namely, those related with PSI (*PsaD*, *PsaE*, *PsaF*, *PsaG*, and *PsaH*; Figure 6). Nevertheless, this downregulation of PSI related genes is likely linked to metabolic adjustments needed for Zn mobilization, since in our experiments Zn biofortification did not negatively affect plant growth and yield (our lab, unpublished data), and because a positive impact on photosynthesis has been frequently reported in Zn fertilized/biofortified cereals (e.g., Lidon et al., 2015, 2017; Liu et al., 2016; Chattha et al., 2017). This is in agreement with the fact that most Zn is taken up by active transport and that the energy demanded by this process is largely supported by photosynthesis (Nakandalage et al., 2016).

In double biofortified plants (Zn and Se), genes enconding a CGS and an UDP-glucuronosyl/UGT were transcriptionally activated. These genes are usually involved in amino acid (methionine) (Zhao et al., 2018) and flavonoid (Yin et al., 2017) biosynthesis, suggesting the activation of these two pathways during double biofortification conditions. CGS is specifically associated with perturbation of the folates pool that serve as donors and acceptors in one-carbon (C1) transfer reactions, which are essential in major metabolic pathways, such as amino acids, nucleic acids, and vitamin B5 (Loizeau et al., 2017). Crops that are able to *de novo* synthesize folates serve as an excellent dietary source, which is not the case of most staple crops, such as rice, potato, and maize (Gorelova et al., 2017). Thus, biofortification with Zn-Se could have a positive impact regarding the enhancement of folate content, further improving the nutritional value of rice, a result that should be tackled in future studies. One DEG (Os03g0103300) from the Se-Zn biofortification was a QLTG-3-1 protein targeted for low-temperature germinability detected on chromosome 3 (Supplementary Table S4), emphasizing the importance of this gene for further molecular characterization of biofortification traits. Improvement of cold tolerance at the germination stage is a major determinant for rice cultivation in tropical or subtropical areas causing severe reductions in the final yield and in crop productivity (Jiang et al., 2017). Further identification of major QTLs associated with the molecular basis of micronutrient uptake/homeostasis (Swamy et al., 2016; Raza et al., 2019) will facilitate the identification of genes of interest and its exploration for breeding Se and Zn rich rice varieties. Six common DEGs were shared between the three biofortified pools, interestingly with similar FC trends in Se and Zn biofortified flag leaves and opposite expression patterns in the Zn-Se pool (Supplementary Tables S2–S4). Among them, eukaryotic initiation factor (eIEF) 3 and eIEF 4 were, respectively, down- and upregulated in the Se (FC −6.528 and 6.860) and Zn (FC −6.452 and 6.813) set, and up- and downregulated in the Zn-Se set (FC 2.378 and −2.869). eIEFs are large protein complexes that participate in translation initiation (Yahalom et al., 2008). As referred above, rice biofortification significantly activate a specific and limited set of DEGs, and, thus, it is expected that the set of genes involved in translation is also limited. This is supported by the expression patterns of a queuine tRNA-ribosyltransferase, i.e., negative FC of −2.077 in Se, −2.245 in Zn and −2.303 in Zn-Se, involved in the biosynthesis of tRNA subunits (Zallot et al., 2014) and known to be inhibited by Zn (Schomburg and Schomburg, 2010). The expression pattern of a Cytochrome P450 (CYP) gene followed also the same trend of the eIEF genes, i.e., FC of 6.860 in Se, 6.813 in Zn and −2.869 in Zn-Se, highlighting its importance in monooxygenation/hydroxylation reactions in biochemical pathways (Wei and Chen, 2018) of Se and Zn biofortified plants. The fact that an ethylene response factor 2 was commonly upregulated in the Zn- and in the Se-biofortified pool (Supplementary Tables S2, S3) may imply that Zn and Se are sensed by the plant as a stress condition, leading to ethylene production, which is similar to what happens in Fe-biofortification (dos Santos et al., 2017). DEGs were randomly distributed among the 12 rice chromosomes being predominant on chromosomes 1, 3, and 4, while few could be mapped on chromosomes 10, 11, and 12. Although more genomic studies are necessary to understand the molecular basis of biofortification across rice chromosomes, a meta-analysis of rice QTLs associated with iron and zinc in grains have already identified 48 meta-QTLs (MQTL) randomly distributed across rice chromosomes, though they are predominant on chromosome 7 (27 QTLs) and scarcely mapped on chromosome 11 (8 QTLs; Raza et al., 2019). Several genes/transcripts (e.g., OsATM3, OsDMAS1, OsFRO2, OsNAS1-3, OsVIT2, OsYSL16, OsZIP3, and OsZIP7) are physically located within or near these MQTL regions and are, as found here, involved in binding, oxidation reduction process, metabolic process, regulation of transcription, and transport (Raza et al., 2019).

## Conclusion

In conclusion, we have in this work settled the foundations to understand genomic changes in the flag leaves of rice plants biofortified with the single and combined use of Zn and Se, which are mainly based on the activation of a limited number of metabolic pathways related to micronutrient mobilization and to the specific functions of Zn (i.e., its enzymatic co-factor/coenzyme function in the biosynthesis of nitrogenous compounds, carboxylic acids, organic acids and amino acids) and Se (vitamin biosynthesis and ion homeostasis). The success of this approach should be followed in future studies to understand how landraces and other rice cultivars respond to biofortification. For that, we are currently analyzing the transcriptome of a biofortified rice landrace and integrating agronomic and molecular analyzes to further study the transcriptional patterns of putatively key biofortification genes during grain development.

## Data Availability Statement

The datasets GENERATED for this study can be found in the NCBI Sequence Read Archive under Bioproject No. PRJNA629980.

## Author Contributions

FR, JR, and FL conceptualized the work. FR, JR, FL, AN, and AR-B performed the experimental design. FR, JR, and AN performed the experimental assays. FR, PB-S, and AR-B performed the laboratory work. FR, IM, ME, BS, and AR-B performed the bioinformatic analysis. FR, IM, JR, and AR-B wrote the manuscript draft. ME and BS performed the critical review of the manuscript. FR, IM, JR, and AR-B were responsible for the final version of the manuscript. All authors contributed to the article and approved the submitted version.

## Conflict of Interest

The authors declare that the research was conducted in the absence of any commercial or financial relationships that could be construed as a potential conflict of interest.

## Footnotes

1 http://rice.plantbiology.msu.edu/

2 https://david.ncifcrf.gov/home.jsp

3 http://www.genome.jp/kegg/

4 http://www.genome.jp/kegg/tool/map_pathway3.html
