# Supplementary material for: Rice Biofortification With Zinc and Selenium: A Transcriptomic Approach to Understand Mineral Accumulation in Flag Leaves
Source: Front Genet. 2020 Jul 7;11:543. doi: 10.3389/fgene.2020.00543 (PMC7359728; doi:10.3389/fgene.2020.00543)
Supplement: Supplementary file 1 [file Data_Sheet_1.docx]

**Figure S1.** Chromosomal distribution of rice DEGs after biofortification with Se (blue), Zn (yellow) and Se-Zn (green). Common DEGs are indicated in black. Mapping of significant DEGs was performed onto the 12 chromosomes of rice using chromosome map tool (<http://viewer.shigen.info/oryzavw/maptool/MapTool.do>). The chromosome numbers are shown on the top and the number of DEGs mapped in each one is shown between brackets. Name of DEGs and their genetic locations are showed on the right-side of each chromosome.

**Figure S2.** Significant differentially expressed genes (DEGs) involved in RNA degradation of Zn- biofortified rice-Mak (P<0.05). Genes significantly up-regulated by Zn-biofortification are shown in yellow boxes. White boxes indicate non-responsive genes.

**Figure S3.** Significant differentially expressed genes (DEGs) involved in the Thiamine metabolism (left) and the Biotin metabolism (right) of Se- biofortified rice-Mak (P<0.01). Genes significantly up-regulated by Zn-biofortification are shown in yellow boxes. White boxes indicate non-responsive genes.
