# Supplementary figures and images for: Rice Biofortification With Zinc and Selenium: A Transcriptomic Approach to Understand Mineral Accumulation in Flag Leaves
Source: Front Genet. 2020 Jul 7;11:543. doi: 10.3389/fgene.2020.00543 (PMC7359728; doi:10.3389/fgene.2020.00543)

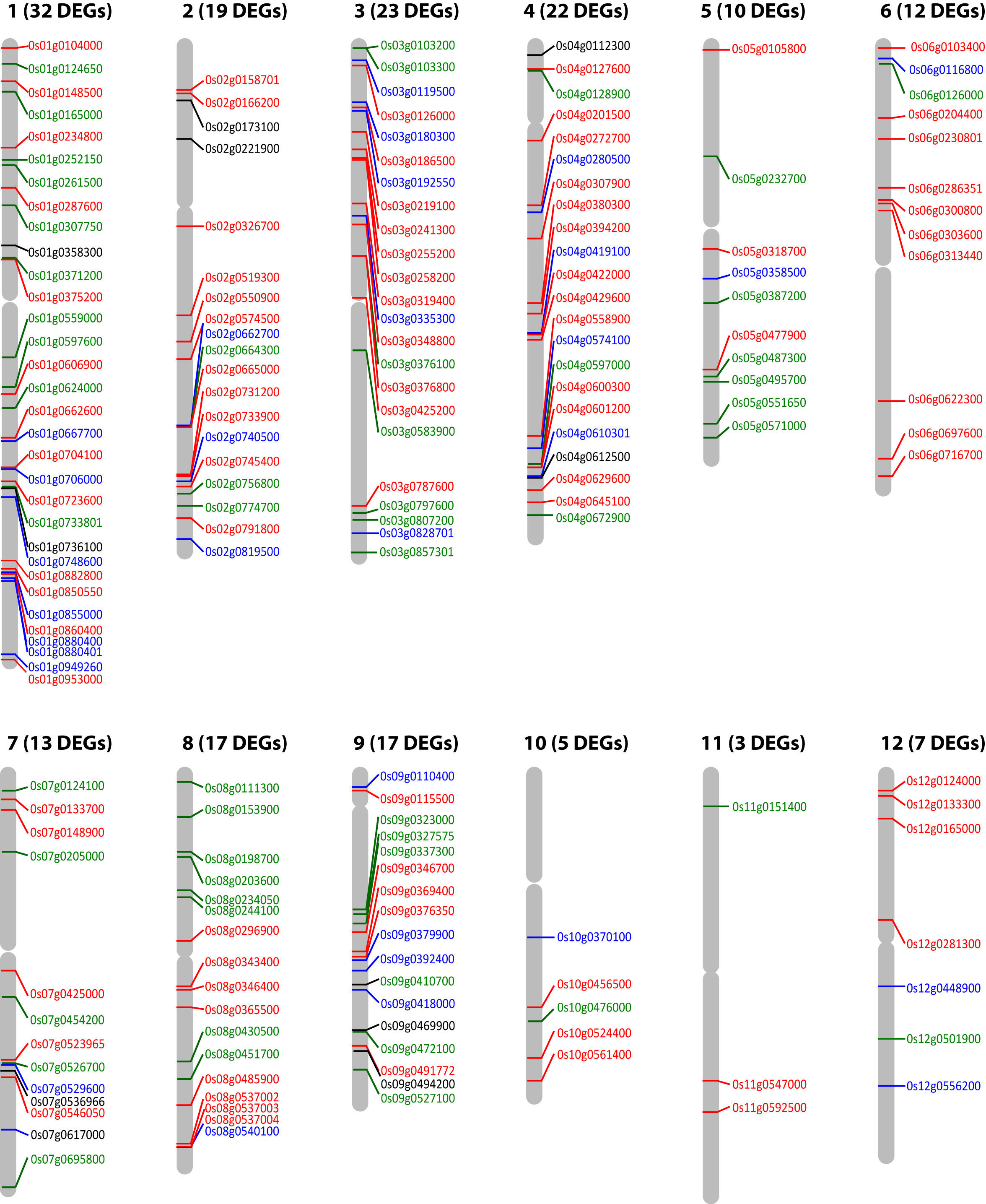

Supplement: Supplementary file 2 [file Image_1.jpg]

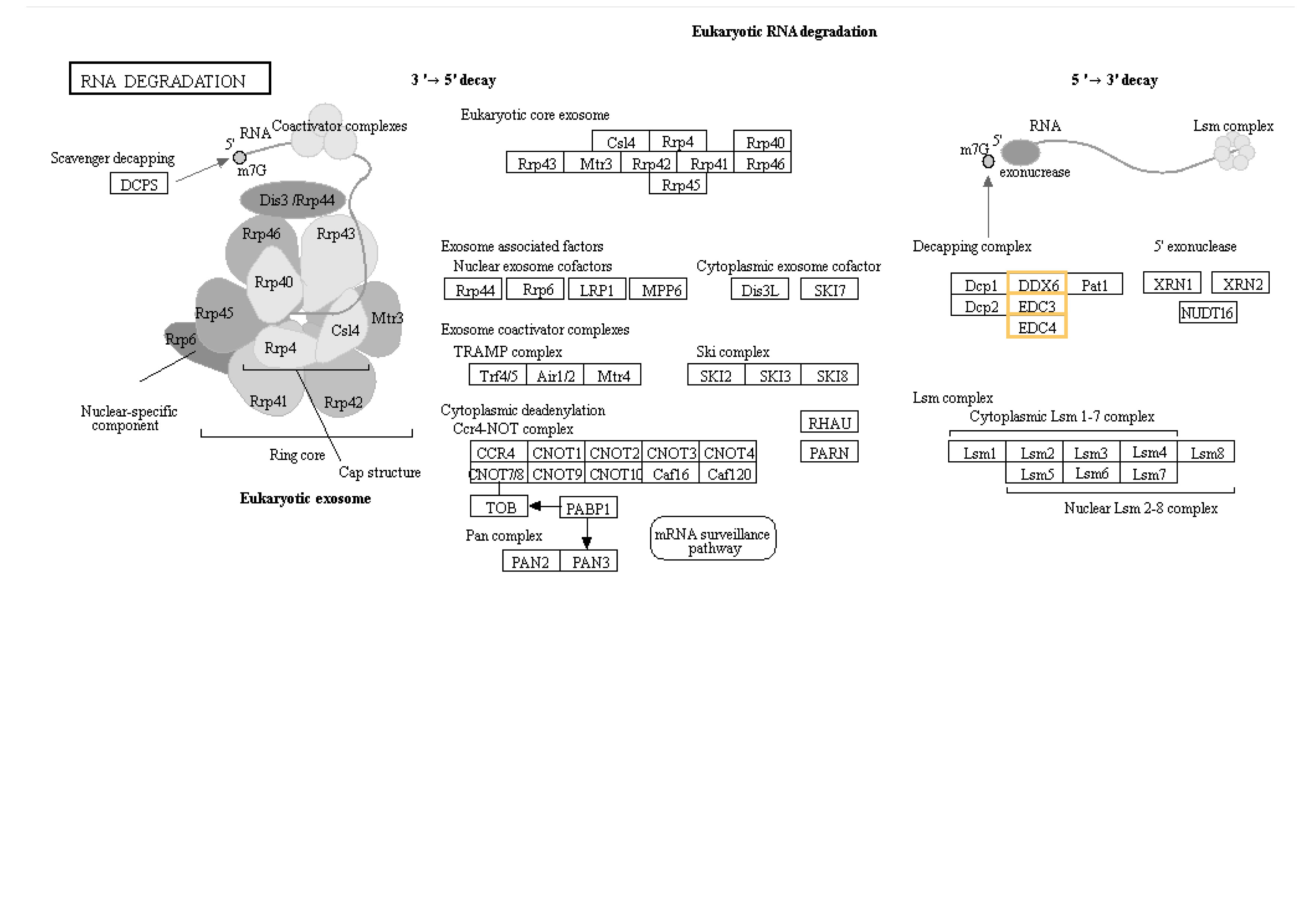

Supplement: Supplementary file 3 [file Image_2.jpg]

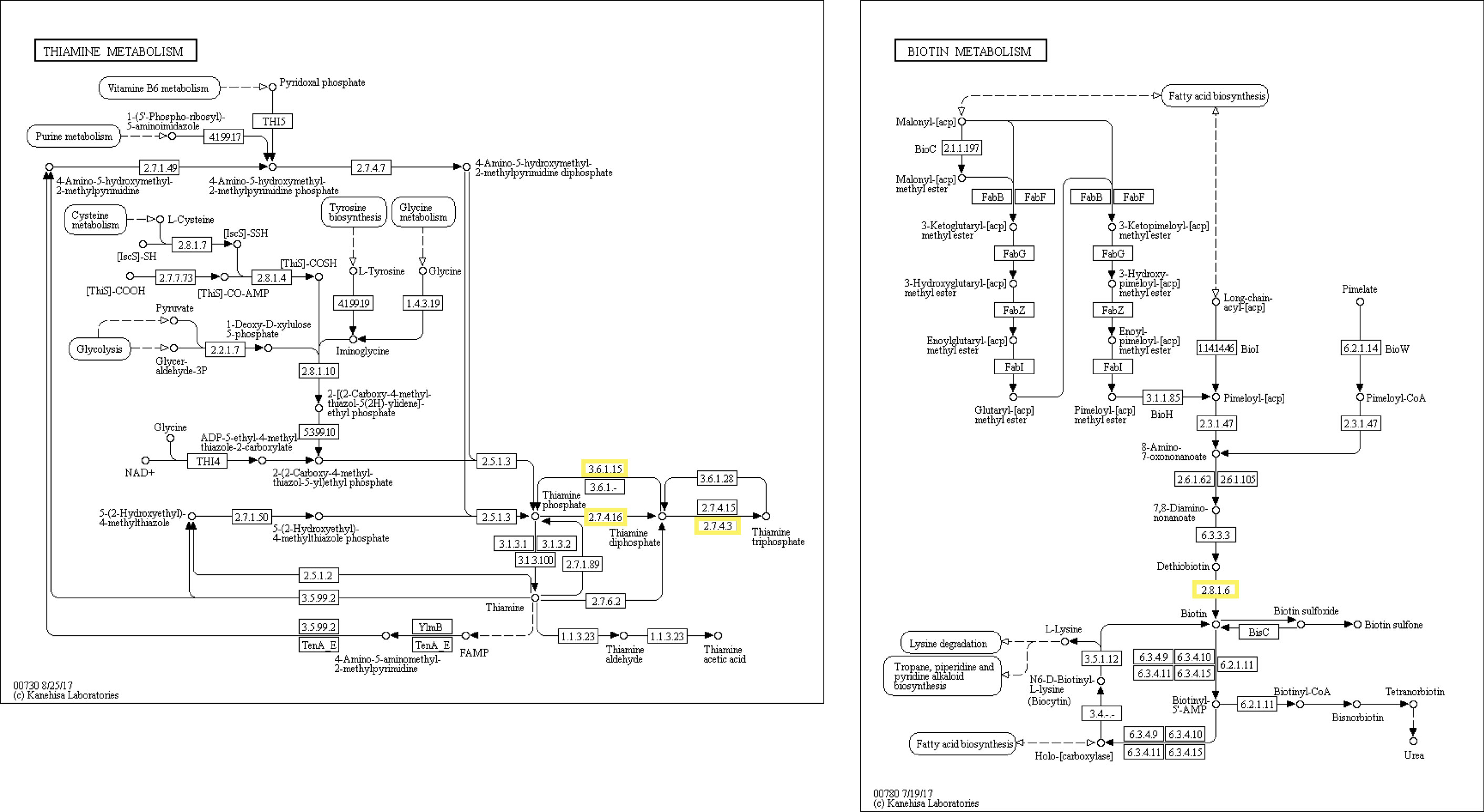

Supplement: Supplementary file 4 [file Image_3.jpg]
