# Supplementary material for: Rice Biofortification With Zinc and Selenium: A Transcriptomic Approach to Understand Mineral Accumulation in Flag Leaves
Source: Front Genet. 2020 Jul 7;11:543. doi: 10.3389/fgene.2020.00543 (PMC7359728; doi:10.3389/fgene.2020.00543)
Supplement: Supplementary file 5 [file Table_1.docx]

**Table S1.** Details of RNA-Seq data of rice cultivar Makassane (Mak) in control conditions (Ctr) and under different treatments of Selenium (Se500: 500 g ha^-1^), Zinc (Zn900: 900 g ha^−1^), and Zn-Se. Numbers indicate the total number of reads after trimming (Total reads), GC content (GC%), number of cleaned reads after trimming (Processed), number of reads mapped to the reference (Mapped) and number of reads that failed to aligned (Unmapped).

| **Sample ID** | **Total reads** | **GC (%)** | **Processed** | **Mapped** | **Unmapped** |
| --- | --- | --- | --- | --- | --- |
| MakCtr-1A | 40,559,462 | 53.96 | 39,954,524 | 37,730,322 (94.43%) | 2,224,202 (5.57%) |
| MakCtr-1B | 34,178,408 | 54.68 | 33,520,796 | 31,034,156 (92.58%) | 2,486,640 (7.42%) |
| MakCtr-1C | 35,727,638 | 53.47 | 35,142,494 | 32,842,616 (93.46%) | 2,299,878 (6.54%) |
| MakSe500-7A | 36,231,872 | 52.53 | 35,629,422 | 33,663,804 (94.48%) | 1,965,618 (5.52%) |
| MakSe500-7B | 36,884,804 | 53.54 | 36,298,796 | 31,940,744 (87.99%) | 4,358,052 (12.01%) |
| MakSe500-7C | 30,238,786 | 52.37 | 29,794,568 | 26,748,968 (89.78%) | 3,045,600 (10.22%) |
| MakZn900-3A | 43,802,954 | 53.14 | 43,093,136 | 39,847,134 (92.47%) | 3,246,002 (7.53%) |
| MakZn900-3B | 39,963,042 | 52.57 | 39,220,998 | 36,207,359 (92.32%) | 3,013,639 (7.68%) |
| MakZn900-3C | 35,700,524 | 53.03 | 35,174,796 | 33,135,233 (94.2%) | 2,039,563 (5.8%) |
| MakZn-Se-9A | 36,031,594 | 53.18 | 35,479,852 | 32,907,538 (92.75%) | 2,572,314 (7.25%) |
| MakZn-Se-9B | 39,418,854 | 53.73 | 38,804,652 | 35,207,467 (90.73%) | 3,597,185 (9.27%) |
| MakZn-Se-9C | 47,758,632 | 53.15 | 46,900,430 | 44,032,295 (93.88%) | 2,868,135 (6.12%) |
