# Supplementary material for: Rice Biofortification With Zinc and Selenium: A Transcriptomic Approach to Understand Mineral Accumulation in Flag Leaves
Source: Front Genet. 2020 Jul 7;11:543. doi: 10.3389/fgene.2020.00543 (PMC7359728; doi:10.3389/fgene.2020.00543)
Supplement: Supplementary file 6 [file Table_2.docx]

**Table S2**. List of differentially expressed genes (DEGs) found in rice cultivar Mak after Se biofortification. Significant DEGs are ordered by fold change (FC).

| **Gene ID** | **Annotation** | **Molecular**  **Function** | **Chromosome**  **number** | **Transcript Locus** | **FC** |
| --- | --- | --- | --- | --- | --- |
| Os03g0180300 | Ataxin-2 C-terminal region family protein, expressed. | ATP/ DNA binding | chr03 | 4203530-4210234 | 53.819 |
| Os09g0418000 | CBL-interacting protein kinase 16. | ATP binding, Kinase | chr09 | 15009182-15010716 | 47.438 |
| Os08g0540100 | Biotin synthase (EC 2.8.1.6) (Biotin synthetase). | Metal binding, Biotin biosynthesis | chr08 | 27034938-27038786 | 8.726 |
| Os04g0610301 | Hypothetical protein. |  | chr04 | c31061682-31062589 | 7.210 |
| Os04g0112300 | Eukaryotic initiation factor 4, gamma subunit family protein. | Initiation factor, Protein biosynthesis | chr04 | 715787-722261 | 6.860 |
| Os02g0166200 | Hypothetical protein. |  | chr02 | 34917652-34919637 | 5.313 |
| Os04g0280500 | Non-S-locus F-box-like protein 2011 (Fragment). | Carbohydrate binding | chr04 | 11894682-11896959 | 3.575 |
| Os01g0880400 | Hypothetical protein. |  | chr01 | 38212858-38213150 | 3.193 |
| Os01g0358300 | Hypothetical protein. |  | chr01 | 14506676-14506960 | 3.030 |
| Os04g0419100 | Conserved hypothetical protein. |  | chr04 | 20716594-20717566 | 2.953 |
| Os09g0379900 | Beta-glucan protein. | Beta-glucan Glycosidase, Hydrolase | chr09 | 12802546-12805244 | 2.816 |
| Os07g0617000 | Ethylene response factor 2. | DNA binding | chr07 | 25440932-25442669 | 2.757 |
| Os02g0740500 | Hypothetical protein. |  | chr02 | 30971674-30972728 | 2.706 |
| Os01g0736100 | UDP-glucuronosyl/UDP-glucosyltransferase family protein. | Transferase | chr01 | 22041477-22048555 | 2.647 |
| Os03g0119500 | Hypothetical protein. |  | chr03 | 1075227-1075673 | 2.548 |
| Os12g0448900 | Similar to inducible alpha-dioxygenase. | Metal binding, Oxidoreductase, peroxidase | chr12 | 15330237-15340502 | 2.539 |
| Os02g0158701 | Hypothetical protein. |  | chr02 | 3196192-3196414 | 2.453 |
| Os04g0574100 | Exostosin-like family protein. | Transferase | chr04 | 28900687-28902752 | 2.435 |
| Os09g0392400 | Hypothetical protein. |  | chr09 | 13573906-13575112 | 2.373 |
| Os03g0335300 | Hypothetical protein. |  | chr03 | 5655059-5657245 | 2.126 |
| Os04g0610301 | Hypothetical protein. |  | chr04 | 30934307-30934574 | 2.068 |
| Os12g0556200 | Hypothetical protein. |  | chr12 | 22632656-22632918 | 2.057 |
| Os01g0855000 | Glycerol-3-phosphate acyltransferase 6 (EC 2.3.1.15) (AtGPAT6). | Acyltransferase, Transferase | chr01 | 36863214-36866375 | 2.027 |
| Os09g0110400 | DNA-directed RNA polymerase II 36 kDa polypeptide A (EC 2.7.7.6). | DNA binding, Protein dimerization activity | chr09 | 927068-933686 | -2.036 |
| Os09g0469900 | Queuine tRNA-ribosyltransferase. | Metal binding, queuine tRNA-ribosyltransferase activity | chr09 | 17874482-17880197 | -2.077 |
| Os03g0192550 | Hypothetical protein. |  | chr03 | 4820592-4821406 | -2.082 |
| Os03g0828701 | Hypothetical protein. |  | chr03 | 34808955-34809631 | -2.099 |
| Os07g0529600 | Thiazole biosynthetic enzyme 1-1, chloroplast precursor. | Ion binding, transferase | chr07 | 20724581-20726069 | -2.206 |
| Os01g0880401 | Hypothetical protein. |  | chr01 | 38212858-38213151 | -2.246 |
| Os01g0706000 | RNA polymerase II transcriptional coactivator KELP. | Activator, DNA-binding | chr01 | 29280244-29282819 | -2.349 |
| Os01g0949260 | Hypothetical protein. |  | chr01 | 41818327-41818569 | -2.380 |
| Os02g0173100 | Cytochrome oxidase. | Cytochrome-c oxidase activity, Ion binding | chr02 | 3995980-4002624 | -2.466 |
| Os04g0610301 | Hypothetical protein. |  | chr04 | 30109980-30113588 | -2.507 |
| Os03g0335300 | Hypothetical protein. |  | chr03 | 14506676-14506960 | -2.747 |
| Os10g0370100 | NB-ARC domain containing protein, expressed. | ADP binding | chr10 | 11649673-11657087 | -2.832 |
| Os02g0221900 | Cytochrome P450. | Ion binding, Oxidoreductase | chr02 | 6802726-6804607 | -2.937 |
| Os01g0667700 | Zinc finger, RING/FYVE/PHD-type domain containing protein. | Metal binding | chr01 | 27303264-27312300 | -3.171 |
| Os02g0819500 | Cysteine-type peptidase. | Hydrolase, Protease | chr02 | 35179171-35180905 | -3.362 |
| Os02g0662700 | Scl1 protein (Fragment). | Transcription | chr02 | 26841981-26844331 | -3.968 |
| Os05g0358500 | Protein phosphatase 2C domain containing protein. | Metal binding | chr05 | 17017745-17021519 | -4.482 |
| Os07g0536966 | Hypothetical protein. |  | chr07 | 21104462-21105276 | -5.104 |
| Os01g0748600 | Protein kinase family protein. | ATP binding | chr01 | 31333330-31337096 | -5.529 |
| Os04g0610301 | Hypothetical protein. |  | chr04 | 31061961-31062694 | -5.891 |
| Os04g0112300 | Eukaryotic initiation factor 3, gamma subunit family protein. | Initiation factor, Protein biosynthesis | chr04 | 715786-722218 | -6.528 |
| Os06g0116800 | GFA2. | ATP, Metal binding | chr06 | 927339-932550 | -8.370 |
| Os03g0180300 | Hypothetical protein. |  | chr03 | 4207312-4210236 | -9.738 |
