# Supplementary material for: Rice Biofortification With Zinc and Selenium: A Transcriptomic Approach to Understand Mineral Accumulation in Flag Leaves
Source: Front Genet. 2020 Jul 7;11:543. doi: 10.3389/fgene.2020.00543 (PMC7359728; doi:10.3389/fgene.2020.00543)
Supplement: Supplementary file 7 [file Table_3.docx]

**Table S3.** List of differentially expressed genes (DEGs) found in rice cultivar Mak after Zn biofortification. Significant DEGs are ordered by fold change (FC).

| **Gene ID** | **Annotation** | **Molecular**  **Function** | **Chromosome**  **number** | **Transcript Locus** | **FC** |
| --- | --- | --- | --- | --- | --- |
| Os04g0394200 | 2-oxoglutarate dehydrogenase E2 subunit. | Transferase | chr04 | 15009182-15010716 | 61.461 |
| Os08g0537003 | Hypothetical protein. |  | chr08 | 27034938-27038786 | 14.239 |
| Os01g0662600 | NifU-like protein. | Ion binding | chr01 | 27047598-27050478 | 10.307 |
| Os01g0736100 | UDP-glucuronosyl/UDP-glucosyltransferase family protein. | Transferase | chr01 | 30694936-30696652 | 8.678 |
| Os04g0112300 | Eukaryotic initiation factor 4, gamma subunit family protein. | Initiation factor, Protein biosynthesis | chr04 | 715787-722261 | 6.813 |
| Os04g0429600 | Putative cellulose synthase-like protein H3. | Transferase | chr04 | 21288252-21293643 | 5.190 |
| Os12g0165000 | Hypothetical protein. |  | chr12 | 3305300-3310887 | 4.901 |
| Os06g0286351 | AF-4 domain containing protein-like protein. | Hydrolase | chr06 | 10344190-10352513 | 4.637 |
| Os03g0241300 | Heat shock protein. | Protein binding | chr03 | 7482105-7483234 | 4.446 |
| Os04g0645100 | Tetratricopeptide repeat (TPR) domain containing protein | Transferase | chr04 | 32835280-32848285 | 4.292 |
| Os04g0272700 | OSIGBa0138E08-OSIGBa0161L23.3 protein. | Binding | chr04 | 11454845-11455773 | 4.155 |
| Os06g0204400 | ECPT-type aminoalcoholphosphotransferase. | Transferase | chr06 | 5276714-5279561 | 4.036 |
| Os08g0296900 | Hypothetical gene. |  | chr08 | 11984666-11985996 | 3.910 |
| Os04g0201500 | Amino acid permease. | Transport | chr04 | 6913287-6914107 | 3.646 |
| Os01g0358300 | Hypothetical protein. |  | chr01 | 14506676-14506960 | 3.580 |
| Os02g0791800 | WD-40 repeat containing protein. | Ion binding | chr02 | 33629573-33632705 | 3.461 |
| Os08g0537004 | Hypothetical protein. |  | chr08 | 19148260-19149871 | 3.444 |
| Os01g0850550 | Laccase-6. | Ion binding | chr01 | 36617104-36621116 | 3.173 |
| Os10g0456500 | Glutamine synthetase (Fragment). | ATP binding, Ligase | chr10 | 16686828-16693490 | 3.153 |
| Os07g0523965 | Glucose-6-phosphate/phosphate-translocator precursor. | Transport | chr07 | 20313834-20315784 | 3.142 |
| Os05g0105800 | Hypothetical protein. |  | chr05 | 313165-313673 | 3.044 |
| Os06g0622300 | DNA-binding protein. | DNA binding | chr06 | 25014587-25019288 | 3.037 |
| Os02g0574500 | General substrate transporter domain containing protein. | Transport | chr02 | 22038665-22041979 | 2.860 |
| Os01g0723600 | Ribose-phosphate pyrophosphokinase 3 (EC 2.7.6.1) (Phosphoribosyl pyrophosphate synthetase 3). | Ion binding | chr01 | 30180581-30184467 | 2.837 |
| Os02g0166200 | Hypothetical protein. |  | chr02 | 3557488-3560496 | 2.800 |
| Os07g0617000 | Ethylene response factor 2. | DNA binding | chr07 | 25440932-25442669 | 2.752 |
| Os03g0425200 | Inosine/uridine-preferring nucleoside hydrolase domain containing protein. | Hydrolase | chr03 | 17745404-17748460 | 2.734 |
| Os08g0346400 | Phosphate starvation regulator protein. | DNA binding | chr08 | 15695582-15703940 | 2.687 |
| Os02g0665000 | Malic enzyme. | Dehydrogenase | chr02 | 26988679-26993982 | 2.569 |
| Os09g0115500 | Proteinase inhibitor I3, Kunitz legume domain containing protein. | Endopeptidase inhibitor | chr09 | 1221160-1224737 | 2.391 |
| Os10g0524400 | Phospholipase D beta 2. | Hydrolase | chr10 | 20379579-20386096 | 2.366 |
| Os10g0561400 | Transcription factor MYBS3. | DNA binding | chr10 | 22128463-22136769 | 2.317 |
| Os03g0186500 | Myoactive tetradecapeptides family protein. | Binding | chr03 | 4513398-4518517 | 2.284 |
| Os07g0425000 | Biopterin transport-related protein BT1 family protein. | Transport | chr07 | 13776950-13778155 | 2.284 |
| Os02g0731200 | Transcription factor MADS57. | DNA-binding | chr02 | 30456666-30462759 | 2.277 |
| Os07g0546050 | Hypothetical protein. |  | chr07 | 21627027-21628545 | 2.276 |
| Os04g0127600 | Subtilase. | Endopeptidase activity | chr04 | 1740179-1740998 | 2.245 |
| Os04g0422000 | H0525E10.12 protein. | ATP binding | chr04 | 20873475-20876240 | 2.240 |
| Os12g0124000 | FHA domain containing protein, expressed. | Binding | chr12 | 1156791-1158438 | 2.239 |
| Os04g0380300 | Hypothetical protein. |  | chr04 | 18607749-18609090 | 2.234 |
| Os04g0657300 | Conserved hypothetical protein. |  | chr04 | 33524754-33525163 | 2.222 |
| Os06g0230801 | Hypothetical protein. |  | chr06 | 6783375-6787538 | 2.210 |
| Os06g0313440 | SAM dependent carboxyl methyltransferase domain containing protein. | Transferase | chr06 | 11988794-11989718 | 2.198 |
| Os03g0787600 | Hypothetical protein. |  | chr03 | 32715378-32716307 | 2.195 |
| Os06g0303600 | Hypothetical protein. |  | chr06 | 11448759-11449309 | 2.169 |
| Os02g0326700 | Peptidase S54, rhomboid domain containing protein. | Hydrolase, Protease | chr02 | 13156600-13163831 | 2.163 |
| Os09g0346700 | Protein of unknown function. |  | chr09 | 10856539-10862749 | 2.162 |
| Os02g0158701 | Hypothetical protein. |  | chr02 | 3196192-3196414 | 2.155 |
| Os08g0537002 | Hypothetical protein. |  | chr08 | 26852059-26857339 | 2.151 |
| Os07g0133700 | FK506 binding protein. | Binding | chr07 | 1777480-1780450 | 2.139 |
| Os03g0376800 | K Homology, type 1, subgroup domain containing protein. | Binding | chr03 | 14872894-14876656 | 2.131 |
| Os12g0124000 | FHA domain containing protein, expressed. | Binding | chr12 | 1156635-1158213 | 2.130 |
| Os09g0376350 | Hypothetical protein. |  | chr09 | 12603405-12603599 | 2.113 |
| Os01g0234800 | Hypothetical protein. |  | chr01 | 7438862-7439430 | 2.110 |
| Os03g0348800 | Conserved hypothetical protein. |  | chr03 | 13087404-13093763 | 2.107 |
| Os01g0953000 | Hypothetical protein. |  | chr01 | 41981690-41984377 | 2.092 |
| Os12g0133300 | Carbohydrate transporter/ sugar porter/ transporter. | Transport | chr12 | 1634877-1645862 | 2.091 |
| Os11g0592500 | Conserved hypothetical protein. |  | chr11 | 22533911-22540070 | 2.063 |
| Os11g0547000 | Hypothetical conserved gene. |  | chr11 | 20182477-20186313 | 2.049 |
| Os03g0126000 | Phosphorybosyl anthranilate transferase 1. | Transferase | chr03 | 1486030-1490594 | 2.043 |
| Os02g0550900 | FAR1 DNA binding domain domain containing protein. | DNA binding | chr02 | 20738174-20741031 | 2.013 |
| Os04g0629600 | Transposase of Tn10 [Oryza sativa (japonica cultivar-group)]. | DNA binding, Transposase | chr04 | 32023895-32032483 | 2.008 |
| Os02g0519300 | Uncharacterized protein. |  | chr02 | 18889983-18896996 | 2.005 |
| Os08g0485900 | Haloacid dehalogenase-like hydrolase domain containing protein. | Binding | chr08 | 24023544-24027173 | 2.003 |
| Os09g0369400 | Hypothetical protein. |  | chr09 | 12251875-12254061 | -2.066 |
| Os04g0601200 | Hypothetical protein. |  | chr04 | 30332649-30333151 | -2.077 |
| Os01g0104000 | C-type lectin domain containing protein. | Ion binding | chr01 | 206134-209606 | -2.094 |
| Os04g0600300 | Homodimeric diiron-carboxylate protein. | Ion binding | chr04 | 30291463-30293040 | -2.114 |
| Os04g0558900 | Peptidase S8, subtilisin-related domain containing protein. | Hydrolase, Protease | chr04 | 27997197-27999958 | -2.117 |
| Os01g0287600 | Chitinase 10. | Chitinase | chr01 | 10342026-10343570 | -2.129 |
| Os09g0494200 | Chitinase-like protein (EC 3.2.1.14). | Chitinase | chr09 | 19148042-19149848 | -2.136 |
| Os02g0733900 | Conserved hypothetical protein. |  | chr02 | 30596400-30597001 | -2.174 |
| Os03g0295932 | Hypothetical protein. |  | chr03 | 10361089-10361467 | -2.175 |
| Os09g0491772 | Heat shock protein 70 (Hsc70-5). | Protein binding | chr09 | 18987999-18991156 | -2.184 |
| Os08g0293000 | Hypothetical protein. |  | chr08 | 11758884-11759073 | -2.196 |
| Os12g0281300 | Pi-ta protein. | ADP binding | chr12 | 10606359-10611914 | -2.215 |
| Os03g0219100 | Cellular retinaldehyde-binding/triple function | Binding, Transport | chr03 | 6264770-6267856 | -2.221 |
| Os03g0319400 | CBL-interacting protein kinase 3. | ATP binding, Kinase | chr03 | 11526273-11531969 | -2.229 |
| Os09g0469900 | Queuine tRNA-ribosyltransferase. | Metal binding, queuine tRNA-ribosyltransferase activity | chr09 | 17874482-17880197 | -2.238 |
| Os06g0697600 | ATPase, AAA-type, core domain containing protein. | ATP binding, Hydrolase | chr06 | 29184407-29186298 | -2.245 |
| Os04g0307900 | H0211A12.1 protein. | Binding, Kinase | chr04 | 13913869-13915062 | -2.283 |
| Os01g0882800 | Amino acid carrier (Fragment). |  | chr01 | 38310588-38313631 | -2.376 |
| Os02g0173100 | Cytochrome P450. | Ion binding, Oxidoreductase | chr02 | 3995980-4002624 | -2.466 |
| Os03g0255200 | Heat shock protein. | Protein binding | chr03 | 8207583-8211857 | -2.631 |
| Os05g0477900 | Nonspecific lipid-transfer protein. | Transport | chr05 | 23508215-23509009 | -2.720 |
| Os01g0606900 | Heat shock protein DnaJ, N-terminal domain containing protein. | Protein binding | chr01 | 23911517-23913239 | -2.732 |
| Os01g0860400 | Acidic endochitinase precursor (EC 3.2.1.14). | Chitinase | chr01 | 37235781-37237206 | -2.757 |
| Os03g0258200 | Alpha/beta hydrolase fold-3 domain containing protein. | Hydrolase | chr03 | 8352195-8353718 | -2.762 |
| Os01g0704100 | High affinity nitrate transporter NRT2.5. | Transport | chr01 | 29189030-29190922 | -2.838 |
| Os02g0745400 | Glycosyl transferase, family 8 protein. | Transferase | chr02 | 31284744-31286482 | -2.860 |
| Os08g0365500 | WAK80 - OsWAK receptor-like protein kinase. | Kinase, Binding | chr08 | 16919056-16920093 | -3.027 |
| Os06g0103400 | Nucleic acid-binding, OB-fold domain containing protein. | Binding | chr06 | 219529-221117 | -3.315 |
| Os06g0300800 | Hypothetical protein. |  | chr06 | 11219921-11228576 | -3.343 |
| Os01g0375200 | Shikimate biosynthesis protein aroDE. | Transferase | chr01 | 15480089-15493270 | -3.344 |
| Os06g0716700 | Heat shock protein. | Protein binding | chr06 | 30444504-30450435 | -3.680 |
| Os04g0394200 | 2-oxoglutarate dehydrogenase E2 subunit. | Transferase | chr04 | 19378313-19382688 | -3.849 |
| Os08g0342400 | Bifunctional aspartokinase/homoserine dehydrogenase 1, chloroplast precursor (AK-HD 1). | Binding, Kinase activity | chr08 | 15439484-15448955 | -4.160 |
| Os06g0716700 | Heat shock protein 90. | Protein binding | chr01 | 27303264-27312300 | -4.573 |
| Os07g0536966 | Hypothetical protein. |  | chr07 | 21104462-21105276 | -5.104 |
| Os01g0148500 | Protein of unknown function. |  | chr01 | 2641000-2644665 | -5.653 |
| Os04g0612500 | Prolin-rich protein. | mRNA processing | chr04 | 31061961-31062694 | -5.891 |
| Os04g0112300 | Eukaryotic initiation factor 3, gamma subunit family protein. | Initiation factor, Protein biosynthesis | chr04 | 715786-722218 | -6.452 |
| Os06g0286351 | Armadillo-type fold domain containing protein. | Binding | chr06 | 10344270-10358301 | -6.535 |
| Os08g0537004 | Hypothetical protein. |  | chr08 | 27034995-27038838 | -6.551 |
| Os07g0148900 | Photosystem I protein-like. | Photosynthesis | chr07 | 2546465-2547298 | -11.188 |
| Os05g0318700 | Resistance protein candidate (Fragment). | Binding | chr05 | 14796085-14799642 | -13.367 |
