# Supplementary material for: Rice Biofortification With Zinc and Selenium: A Transcriptomic Approach to Understand Mineral Accumulation in Flag Leaves
Source: Front Genet. 2020 Jul 7;11:543. doi: 10.3389/fgene.2020.00543 (PMC7359728; doi:10.3389/fgene.2020.00543)
Supplement: Supplementary file 8 [file Table_4.docx]

**Table S4.** List of differentially expressed genes (DEGs) found in rice cultivar Mak after Se-Zn biofortification. Significant DEGs are ordered by fold change (FC).

| **Gene ID** | **Annotation** | **Molecular**  **Function** | **Chromosome**  **number** | **Transcript Locus** | **FC** |
| --- | --- | --- | --- | --- | --- |
| Os03g0376100 | Cystathionine gamma-synthase (Fragment). | Lyase | chr03 | 14850657-14852107 | 27.268 |
| Os01g0736100 | UDP-glucuronosyl/UDP-glucosyltransferase family protein. | Transferase | chr01 | 30694936-30696652 | 16.969 |
| Os10g0476000 | Adaptin ear-binding coat-associated protein 2. | Transport | chr10 | 17726960-17729492 | 7.718 |
| Os04g0672900 | H0322F07.6 protein. | Transcription | chr04 | 34342695-34345315 | 7.198 |
| Os06g0126000 | Hypothetical conserved gene. |  | chr06 | 1389403-1395801 | 6.862 |
| Os03g0857301 | Hypothetical protein. |  | chr03 | 34917652-34919637 | 6.566 |
| Os01g0124650 | Hypothetical conserved gene. |  | chr01 | 1365586-1366432 | 5.398 |
| Os01g0165000 | DRE binding protein 1. | DNA binding | chr01 | 3356461-3361204 | 4.862 |
| Os03g0103200 | Flavin monooxygenase-like enzyme. | Monooxygenase, Oxidoreductase | chr03 | 211082-211817 | 4.720 |
| Os08g0234050 | Conserved hypothetical protein. |  | chr08 | 8154564-8155536 | 4.662 |
| Os03g0583900 | Endoribonuclease Dicer homolog 2a. | Metal binding | chr03 | 21500778-21507797 | 4.391 |
| Os01g0559000 | Thioredoxin family Trp26. | Metal binding | chr01 | 21167915-21172622 | 3.721 |
| Os09g0527100 | RNA-binding protein. | RNA binding | chr09 | 25440932-25442669 | 3.469 |
| Os02g0664300 | Predicted protein. |  | chr02 | 26953166-26965800 | 3.243 |
| Os03g0103300 | QLTG-3-1 protein. | QTL for low-temperature germinability 3-1 | chr03 | 220396-220853 | 3.170 |
| Os09g0527100 | Hypothetical conserved gene. |  | chr09 | 20613400-20625729 | 3.167 |
| Os08g0153900 | Protein of unknown function DUF599 family protein. | Translation | chr08 | 3093804-3094878 | 2.998 |
| Os11g0151400 | Cytochrome family protein. | Ion binding, Oxidoreductase | chr11 | 2390496-2392366 | 2.954 |
| Os01g0597600 | Amino acid transporter, transmembrane domain containing protein. | Transport | chr01 | 23447173-23448687 | 2.771 |
| Os07g0205000 | Ubiquinol-cytochrome c reductase complex 14 kDa protein. | Electron transport | chr07 | 28939652-28940925 | 2.752 |
| Os09g0323000 | UDP-D-glucose epimerase 2. | Isomerase, binding | chr09 | 9437673-9438787 | 2.719 |
| Os08g0203600 | Hypothetical conserved gene. |  | chr08 | 6032289-6039069 | 2.595 |
| Os05g0495700 | Glycerol-3-phosphate dehydrogenase. | Oxidoreductase | chr05 | 24357908-24361112 | 2.552 |
| Os02g0756800 | Phosphate-induced protein 1 conserved region family protein. | DNA binding | chr02 | 31840547-31841768 | 2.475 |
| Os05g0571000 | SPA1 (SUPPRESSOR OF PHYA-105 1)%3B protein binding / signal transducer. | ATP binding, Protein kinase | chr05 | 28445224-28451170 | 2.463 |
| Os01g0733801 | Hypothetical protein. |  | chr01 | 30600797-30602030 | 2.433 |
| Os05g0487300 | Conserved hypothetical protein. |  | chr05 | 23978676-23979669 | 2.386 |
| Os04g0112300 | Eukaryotic initiation factor 3, gamma subunit family protein. | Initiation factor, Protein biosynthesis | chr04 | 11984666-11985996 | 2.378 |
| Os05g0387200 | UDP-sulfoquinovose synthase. | Hydrolase, Coenzyme binding | chr05 | 18738656-18741959 | 2.278 |
| Os07g0124100 | Phytosulfokines 4 precursor. | Developmental protein, Growth factor | chr07 | 1255047-1255744 | 2.174 |
| Os02g0664300 | Predicted protein. |  | chr02 | 27844656-27847779 | 2.154 |
| Os12g0501900 | Uncharacterized protein. |  | chr12 | 19119356-19122575 | 2.149 |
| Os08g0198700 | Glycolate oxidase (EC 1.1.3.15) (Fragment). | Oxidoreductase, Binding | chr08 | 5691931-5697423 | 2.138 |
| Os03g0807200 | Hypothetical protein. |  | chr03 | 33737458-33738155 | 2.109 |
| Os01g0252150 | Hypothetical protein. |  | chr01 | 8331441-8333267 | 2.070 |
| Os01g0307750 | Hypothetical gene. |  | chr01 | 11515321-11515989 | 2.056 |
| Os08g0111300 | Transferase family protein. | Transferase | chr08 | 594453-598038 | 2.055 |
| Os01g0371200 | Glutathione-S-transferase 19E50. | Transferase | chr01 | 15281170-15283073 | 2.041 |
| Os09g0472100 | ABC transporter. | Transport, ATP binding | chr09 | 18034121-18039233 | 2.001 |
| Os01g0261500 | NAD(P)-binding domain containing protein. | Binding | chr01 | 8810826-8812972 | -2.009 |
| Os08g0244100 | Syntaxin 6, N-terminal domain containing protein. | Transport | chr08 | 8803165-8806506 | -2.011 |
| Os05g0232700 | Conserved hypothetical protein. |  | chr05 | 8046789-8047587 | -2.038 |
| Os07g0454200 | Hypothetical protein. |  | chr07 | 15726341-15728745 | -2.065 |
| Os05g0551650 | Hypothetical protein. |  | chr05 | 27422900-27424575 | -2.068 |
| Os08g0451700 | Conserved hypothetical protein. |  | chr08 | 19494586-19497282 | -2.071 |
| Os03g0797600 | BHLH transcription factor. | DNA binding | chr03 | 33233107-33236885 | -2.093 |
| Os04g0601200 | Hypothetical protein. |  | chr04 | 30332649-30333151 | -2.093 |
| Os02g0774700 | Conserved hypothetical protein. |  | chr02 | 32700433-32704631 | -2.128 |
| Os02g0173100 | Cytochrome P450. | Ion binding, Oxidoreductase | chr02 | chr02:3995980-4002624 | -2.192 |
| Os04g0128900 | Flavin monooxygenase-like enzyme, Auxin biosynthesis. | Monooxygenase, Oxidoreductase | chr04 | 1813960-1816070 | -2.237 |
| Os03g0376100 | Cystathionine gamma-synthase (Fragment). | Transferase | chr03 | 14850286-14852220 | -2.252 |
| Os08g0451700 | Conserved hypothetical protein. |  | chr08 | 22084917-22093757 | -2.278 |
| Os09g0410700 | Helix-loop-helix DNA-binding domain containing protein. | DNA binding | chr09 | 14583538-14586281 | -2.292 |
| Os09g0469900 | Queuine tRNA-ribosyltransferase. | Metal binding, queuine tRNA-ribosyltransferase activity | chr09 | 17874482-17880197 | -2.303 |
| Os07g0526700 | Conserved hypothetical protein. |  | chr07 | 20535857-20536812 | -2.492 |
| Os01g0624000 | Neutral ceramidase. | Ceramidase, Hydrolase | chr01 | 24913567-24919962 | -2.530 |
| Os09g0494200 | Chitinase-like protein (EC 3.2.1.14). | Glycosidase, Hydrolase | chr09 | 28488022-28488642 | -2.718 |
| Os08g0293000 | Hypothetical protein. |  | chr08 | 11758884-11759073 | -2.719 |
| Os10g0476000 | Adaptin ear-binding coat-associated protein 1 NECAP-1 family protein. | Transport | chr10 | 17726512-17729548 | -2.758 |
| Os04g0597000 | Secretory carrier membrane protein. | Transport | chr04 | 30109980-30113588 | -2.773 |
| Os03g0857301 | Hypothetical protein. |  | chr03 | 36177802-36178475 | -2.853 |
| Os04g0112300 | Eukaryotic initiation factor 4, gamma subunit family protein. | Initiation factor, Protein biosynthesis | chr06 | 11219921-11228576 | -2.869 |
| Os09g0527100 | RNA-binding protein. | Binding | chr09 | 20613388-20625723 | -3.283 |
| Os03g0376100 | Cystathionine gamma-synthase (Fragment). | Transferase | chr03 | 14850286-14852220 | -3.405 |
| Os09g0337300 | Hypothetical gene. |  | chr09 | 10252139-10252775 | -3.545 |
| Os08g0430500 | 14-3-3-like protein GF14-C. | Binding | chr08 | 20812503-20814404 | -3.604 |
| Os07g0695800 | OSIGBa0096P03.7 protein. | Binding | chr07 | 29658998-29662462 | -3.605 |
| Os09g0327575 | Pi5-1. | ADP Binding | chr09 | 9667216-9670256 | -3.882 |
| Os01g0165000 | Hypothetical gene. |  | chr01 | 3356383-3358426 | -4.850 |
| Os04g0672900 | H0322F07.6 protein. | Transcription | chr04 | 34342607-34345299 | -5.391 |
| Os04g0612500 | Prolin-rich protein. | mRNA processing | chr04 | 31061961-31062694 | -5.896 |
| Os01g0733801 | DRE binding protein 2. | DNA binding | chr01 | 30694998-30696704 | -13.943 |
